# Transforming Growth Factor-β1 and -β2 in Gastric Precancer and Cancer and Roles in Tumor-Cell Interactions with Peripheral Blood Mononuclear Cells *In Vitro*

**DOI:** 10.1371/journal.pone.0054249

**Published:** 2013-01-14

**Authors:** Gui-Fen Ma, Qing Miao, Xiao-Qing Zeng, Tian-Cheng Luo, Li-Li Ma, Yi-Mei Liu, Jing-Jing Lian, Hong Gao, Shi-Yao Chen

**Affiliations:** 1 Department of Gastroenterology, Zhongshan Hospital, Fudan University, Shanghai, China; 2 Endoscopy Center, Zhongshan Hospital, Fudan University, Shanghai, China; Institute of Medical Science, University of Tokyo, Japan

## Abstract

Transforming growth factor-β1 (TGF-β1) and -β2 are correlated with poorer prognosis in gastric cancer (GC), which act in both tumor and immune cells. However, their expressions in precancer and tumor-cell interactions with peripheral blood mononuclear cells (PBMCs) remain unclear. Protein levels of TGF-β1 and -β2 were analyzed by immunohistochemistry and corresponding mRNA levels were determined by quantitative real-time polymerase chain reaction in 93 surgical and biopsy specimens. Serum TGF-β concentration was detected by enzyme-linked immunosorbent assays. AGS and MKN45 cell lines were directly or indirectly cocultured with PBMCs *in vitro*. TGF-β and Smad molecules were detected after cocultures and the growths of GC cells and PBMCs were assessed by cell proliferation assay. The results showed positive staining for TGF-β1 was detected in 20% of control samples, 52.3% of precancer, 59.1% of early GC and 66.7% of advanced GC samples, correlated with lesion progression (χ^2^ = 9.487, *P* = 0.002). All tissues were positive for TGF-β2. TGF-β1 mRNA levels were increased in advanced cancers, while TGF-β2 increased earlier. TGF-β1 mRNA levels were higher in tumor than in peritumor, which positively correlated with Smad2 and Smad7. Serum TGF-β levels were significantly higher in patients with early and advanced cancers compared to controls (TGF-β1∶50.08±4.38 and 45.76±5.00 vs. 27.78±6.11 ng/mL; TGF-β2∶133.61±21.90 and 111.34±15.76 vs. 59.41±15.42 ng/mL, both *P*<0.05). The levels of TGF-β1 mRNA and cytokine secretion were higher in GC cells after direct coculture compared to indirect culture. TGF-β1 was decreased and TGF-β2 was increased in PBMCs after cocultures. Moreover, TGF-β1 inhibited the viability of PBMCs but not cancer cells. Collectively, neoplastic transformation may be an early event involving the increase of TGF-β1 in the general and local environment. TGF-β1 production is promoted by the direct interaction between GC cells and PBMCs, which might facilitate cancer development.

## Introduction

Gastric cancer (GC) is one of the most devastating human cancers, with a highest incidence rate occurring in Eastern Asia [1]. Transforming growth factor β (TGF-β) plays important roles in malignant tumor progression [2]–[4]. The TGF-β family includes TGF-β1, TGF-β2, and TGF-β3, which exhibit different and non-overlapping actions in vitro [5]. TGF-β1 and TGF-β2 mostly contribute to cancer progression by acting in both tumor cells and stromal cells [6], [7], and a loss of sensitivity to growth inhibition by TGF-β is thought to occur in most cancer cells. Meanwhile, cancer cells gain an advantage by selective reduction of the tumor-suppressive activity of TGF-β and augmentation of its oncogenic activity [8], [9]. Previous studies have shown that TGF-β1 constitutes an independent prognostic factor correlated with tumor stage and poorer prognosis [5], 10,11. However, the statuses of TGF-β protein and mRNA and their roles in the transformation from gastric precancer (PC) to carcinoma remain unclear.

TGF-β is a strong immunosuppressive cytokine produced by immune and non-immune cells, including tumor cells [12], [13]. TGF-β may promote tumor growth by inducing epithelial cells to undergo epithelial-mesenchymal transition [14]. Inhibition of TGF-β signaling has been reported to prevent progression and metastasis of certain advanced tumors [15], [16], while TGF-β1 has been shown to reduce the immune response [17], [18] and stimulate angiogenesis [19] in tumor microenvironment. Smad proteins, as intracellular effectors of TGF- β signaling, are activated by receptors and translocate into the nucleus to regulate transcription [20]. However, the Smad-dependence of TGF-β signaling in gastric PC and early cancer is still not fully understood.

TGF-β plays important roles in tumor microenvironment, involving not only interactions among immune and non-immune cells, but also alternation of some cytokines production. Peripheral blood mononuclear cells (PBMCs) are key cytokine-secreting immune cells, and their interactions with cancer cells may induce or suppress cancer-specific immune responses, including apoptosis induction and cytokine production, which contributing mostly to tumor progression [12], [21], [22]. Interactions between cancer cells and PBMCs occur in two main ways: through direct cell-to-cell contact, and through indirect cytokine-dependent mean. Although some studies have shown that several tumor cells can generate CD4+CD25+ regulatory T cells from peripheral CD4+ naïve T cells through the secretion of TGF-β [23], [24], [25], other has demonstrated that the levels of the cytokines of TNF-α, interleukin (IL)-1β, IFN-γ were increased during the interaction between colon cancer cells and lymphocytes [26]. However, the two methods of contact were not compared in these studies, and the main type of interaction thus remains unknown.

In this study, we evaluated the protein and mRNA levels of TGF-β1, TGF-β2, and other correlated molecules in surgical and endoscopic specimens from patients with precancer and cancer, to analyze their roles in carcinogenesis. We also cocultured GC cells with PBMCs to determine if they interacted through direct cell-to-cell contact-dependent or indirect cytokine-dependent means in a simulated tumor microenvironment.

## Materials and Methods

### Patient Samples

A total of 93 cases were included in this study, comprising 30 surgically resected primary GC specimens, 43 neoplastic and cancerous specimens obtained from endoscopic submucosal dissection (ESD), and 20 control biopsy samples from normal-appearing gastric mucosa in patients free from neoplastic or inflammatory diseases. Characteristics of the patients were analyzed as follows: 20 normal tissues (12 males, 8 females; mean age = 45.20±14.01 years, rang 28–63 years), 21 PC including mainly low-grade or high-grade intraepithelial neoplasia (15 males, 6 females; mean age = 65.86±7.81 years, range 57–79 years), 22 early GC (EGC) defined as superficial tumor invading no more than submucosa (14 males, 7 females; mean age = 63.50±13.82 years, range 41–81 years), and 30 advanced GC (AGC) (21 males, 9 females; mean age = 59.48±10.75 years, range 30–70 years). All the patients were confirmed by pathological examination. Histological type was assessed according to the World Health Organisation classification [27]. The groups studied were demographically comparable to the control group (*P*>0.05).

### Ethics Statement

Patients who received radiochemotherapy, suffered from other cancers, or who had a family history of GC were excluded from the study. Written informed consent was obtained from all the subjects. The project was approved by the Research Ethics Committee of Zhongshan Hospital [28].

### Immunohistochemistry (IHC)

TGF-β1 and TGF-β2 protein levels were examined by IHC in 4-µm-thick paraffin sections cut from a single selected block containing neoplastic and non-neoplastic gastric tissues. Samples were routinely dewaxed and hydrated. After blocking of endogenous peroxidase activity, antigens were retrieved by heating with ethylenediamine tetraacetic acid (pH = 9.0). Antigens were subsequently detected using a standard staining procedure (EnVision™ Detection Kit, Dako, CA, USA). Rabbit polyclonal antibodies were used to detect TGF-β1 and TGF-β2 (all dilutions 1∶100; Santa Cruz Biotechnology, CA). For antibody-negative controls, the primary antibodies were substituted with normal rabbit serum. Cases were regarded as positive if at least 5% of dysplastic or cancer cells displayed cytoplasmic staining for TGF-β1 or TGF-β2 at ×100 magnification.

### Quantitative Real-time Polymerase Chain Reaction (qRT-PCR)

Total RNA was isolated from biopsy and surgical specimens, or from cultured cells, using Trizol reagent (Invitrogen, USA). Complementary DNA was prepared using oligo^dT^ primers according to the protocol supplied with the Primer Script ™ RT Reagent (TaKaRa, Tokyo, Japan). Expression levels of TGF-β1, TGF-β2, Smad2, Smad3, Smad4 and Smad7 mRNAs were confirmed by SYBR® Green II qRT-PCR using Mastercycler ep realplex (Eppendorf, Hamburg,Germany) with two-step, at 95°C for 30 seconds then 60°C for 1 min, repeated for 40 cycles. Aliquots of the PCR products were analyzed by melting curves to test their specificity. All the primers, including TGF-β1, TGF-β2, Smad2, Smad3, Smad4 and Smad7, were tested for amplification efficiency and normalized to the mRNA levels of glyceraldehyde-3-phosphate dehydrogenase (GAPDH) (Table S1). All qRT-PCR experiments were performed by the same investigator with no knowledge of the corresponding clinical data.

### Cells and Cell Culture

AGS and MKN45 GC cell lines were purchased from Shanghai Institute of Cell Biology, Chinese Academy of Sciences (Shanghai, China). They were routinely cultured in DMEM medium (Gibco, Invitrogen, USA) supplemented with 10% foetal bovine serum (FBS), 100 U/mL penicillin and 100 ug/mL streptomycin (Gibco) in 5% CO_2_ incubator at 37°C.

### Isolation of PBMCs

PBMCs were isolated from venous blood of GC patients or controls, as described previously [22], [29]. Briefly, 3 mL of blood were immediately diluted in 3 mL of phosphate-buffered saline and layered on 3 mL of Ficoll-Paque Plus™ (Amersham Healthcare, Aylesbury, UK). After centrifugation, PBMCs were recovered from the interphase layer, resuspended in complete culture medium and cultured at 37°C for 24 h to allow attachment of adherent cells, such as dendritic cells.

### Cell Coculture Model

Transwell plates (Corning, New York, USA) were used as an indirect coculture model, which contain bottom chambers and top chambers with 0.4-µm membrane filter pores that do not allow GC cells to pass through but allow medium to exchange freely. Co-incubation of the two types of cells was used as a direct coculture model. Single culture of GC cells was defined as mono-culture. GC cells were adjusted to 5×10^5^ cells/mL, seeded in the bottom chambers of 6-well plates and incubated for 8 h to allow attachment. Inserts containing 5×10^5^ cells/mL of cultured PBMCs were then transferred to the top chambers and cocultured for another 24 h in FBS-free conditional medium or complete medium. As negative controls, inserts with PBMCs were placed on wells with the same culture medium in the absence of cancer cells, and wells with GC cells were left without inserts. The cell count in the monoculture group was double that in the coculture group, to ensure similar cell numbers in all groups. Supernatants and cells were collected separately after 24 h for further use.

### Cell Proliferation Assay

A Cell-IQ cell culturing platform (Chip-Man Technologies, Tampere, Finland), equipped with a phase-contrast microscope (Nikon CFI Achromat phase contrast objective with ×10 magnification, Nikon, Japan) and a camera, was used to detect the growth of tumor cells, as described previously [30]. Briefly, GC cells were cultured on 24-well plates (1×10^4^ cells/well) for 24 h and then treated with TGF-β1 (Peprotech, USA) at 25 ng/mL. Control groups were left untreated. Cells were then incubated for a further 72 h in the Cell-IQ system. Images were captured at 30-min intervals for 72 h, controlled by Image software (Chip-Man Technologies), and analyzed using freely-distributed image software (McMaster Biophotonics Facility, Hamilton, ON), using the manual tracking plug-in created by Fabrice Cordeliéres (Institut Curie, Orsay, France). The Cell-IQ system automatically discriminates the dividing and stable cell stages, and calculates the total cell numbers during proliferation. Eight images were analyzed for each group.

The mobility of lymphocytes makes it difficult to monitor and calculate cell numbers accurately using the Cell-IQ system. We therefore used a Cell Counting Kit-8 (CCK-8) assay (Dojindo, Kumamoto, Japan) to assess the viability of PBMCs, according to the supplier’s instructions. Briefly, cultured PBMCs were seeded at 5×10^3^ cells/well on 96-well plates. Cultures were treated with TGF-β1 or left untreated as controls. After 72 h, CCK-8 reagents were added to each well and the plates were incubated for 4 h. Cell counts were then determined for five wells per experimental group, based on the absorbance at 450 nm of the reduced CCK-8 reagent, using an automicroplate reader (Flexstation 3, Molecular Devices, USA). Cell viability was expressed as the percentage of viable cells relative to the counts of untreated cells. Each experiment was conducted twice. Data were averaged and one representative experiment was shown.

### Enzyme-linked Immunosorbent Assay (ELISA)

TGF-β1 and TGF-β2 levels in monoculture and coculture systems were determined by sandwich ELISA using Quantikine human TGF-β1 immunoassay and TGF-β2 immunoassays (R&D Systems, USA), according to the manufacturer’s instructions. A total of 100 µL of cell supernatants from the direct and indirect culture groups, respectively, were treated with 20 µL of 1 M Hcl for 10 min, followed by neutralization with 20 µL of 1.2 M NaOH. The samples were then pipetted into microplate wells precoated with a monoclonal antibody specific for TGF-β1, and incubated for 2 h at room temperature. An enzyme-linked polyclonal antibody specific for TGF-β1 was then added to the wells and incubated for a further 2 h to sandwich the TGF-β1 ligand. A substrate solution consisting of hydrogen peroxide and tetramethyl benzidine was added and the intensity of the color was determined using an automicroplate reader (Flexstation 3, Molecular Devices). Each experiment was conducted twice and each sample point was assessed in triplicate, and data were averaged.

### Statistical Analysis

Statistical analysis was conducted using SPSS 16.0 for Windows (SPSS, Chicago, USA). Data were presented as means ± SD. Chi-Square tests were used to analyze the correlation between TGF-β staining and clinical pathologic features. Kruskall-Wallis tests were used to compare values among different groups, and Mann-Whitney tests were used to identify specific differences between two groups using a corrected α value. Paired Wilcoxon signed rank tests were performed to compare mRNA levels in tumoral and peritumoral tissues. Concentrations of TGF-β in serum and cell supernatant were analyzed by ANOVA. Bivariate correlation analysis was conducted to examine the associations between mRNA statuses of TGF-β1, TGF-β2, and Smads molecules.

## Results

### Changes in TGF-β1 and TGF-β2 Expression in Dysplasia-carcinoma Sequence

**Figure 1:**
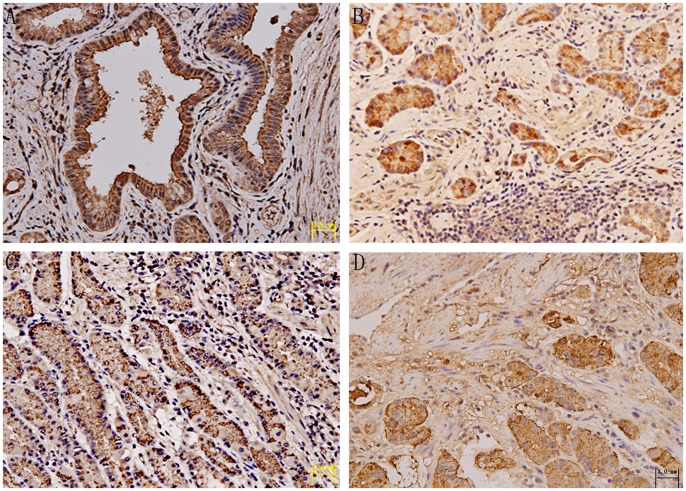
TGF-β1 protein expression in gastric precancer and cancer. (A) Positive staining for TGF-β1 in a case of gastric precancer. (B) Strong cytoplasmic staining in tumor cells limited to the mucosa in a case of early gastric cancer and weak staining in some stromal cells. (C) TGF-β1 expression in a case of intestinal-type advanced gastric cancer, according to Lauren’s classification. (D) Cytoplasmic staining for TGF-β2 in a case of advanced gastric cancer. (All photos are shown at ×200 magnification).

Positive TGF-β1 was present not only in dysplastic or malignant epithelial cells at the top of the gland, adjacent to the lumen, but also strongly in smooth muscle actin expressing fibroblasts (Figure 1A–1C). Positive staining for the intracellular form of TGF-β1 occurred in 20% of the control samples, 52.3% of PC, 59.1% of EGC, and 66.7% of AGC samples. Linear tendency test showed that positive immunostaining rates for TGF-β1 were positively correlated with lesion progression (χ^2^ = 9.487, *P* = 0.002). The histology of GC was then divided into ‘intestinal’ and ‘diffuse’ types, according to the criteria of Lauren [31]. Among the GC samples, 64.1% of intestinal-type showed strong immune reactivity and 63.6% of the diffuse-type were weakly stained. All tissues were stained positive for TGF-β2 (Figure 1D). There was no difference in the expression of TGF-β1 in relation to Helicobacter pylori (*Hp)* infection, Lauren’s classification or lymph node involvement (Table 1).

**Table 1:** Immunoreactivity of TGF-β1 in gastric tissues of precancer and cancer.

Parameter	*n*	No. of positive stains (*n*, %)	No. of negative stains (*n*, %)	*P* value
Different stage of disease				
Control	20	4(20.0)	16(80.0)	0.002
Precancer	21	11(52.3)	10(47.7)	
Early GC	22	13(59.1)	9(40.9)	
Advanced GC	30	20(66.7)*	10(33.3)	
*Hp* infection				
Positive	24	16(66.7)	8(33.3)	0.087
Negative	69	32(46.4)	37(53.6)	
Lauren’s classification				
Intestinal-type	39	25(64.1)	14(35.9)	0.560
Diffuse-type	13	8(63.6)	5(36.4)	
Lymph node involvement				
Positive	27	20(74.1)	7(25.9)	0.099
Negative	25	13(52.0)	12(48.0)	

Data were analyzed by Chi-Square tests.

* , significantly different from controls (*P*<0.05). GC: gastric cancer; *Hp*:Helicobacter pylori.

### Changes in TGF-β1 and TGF-β2 mRNA in Gastric PC and Cancer Tissues

**Figure 2:**
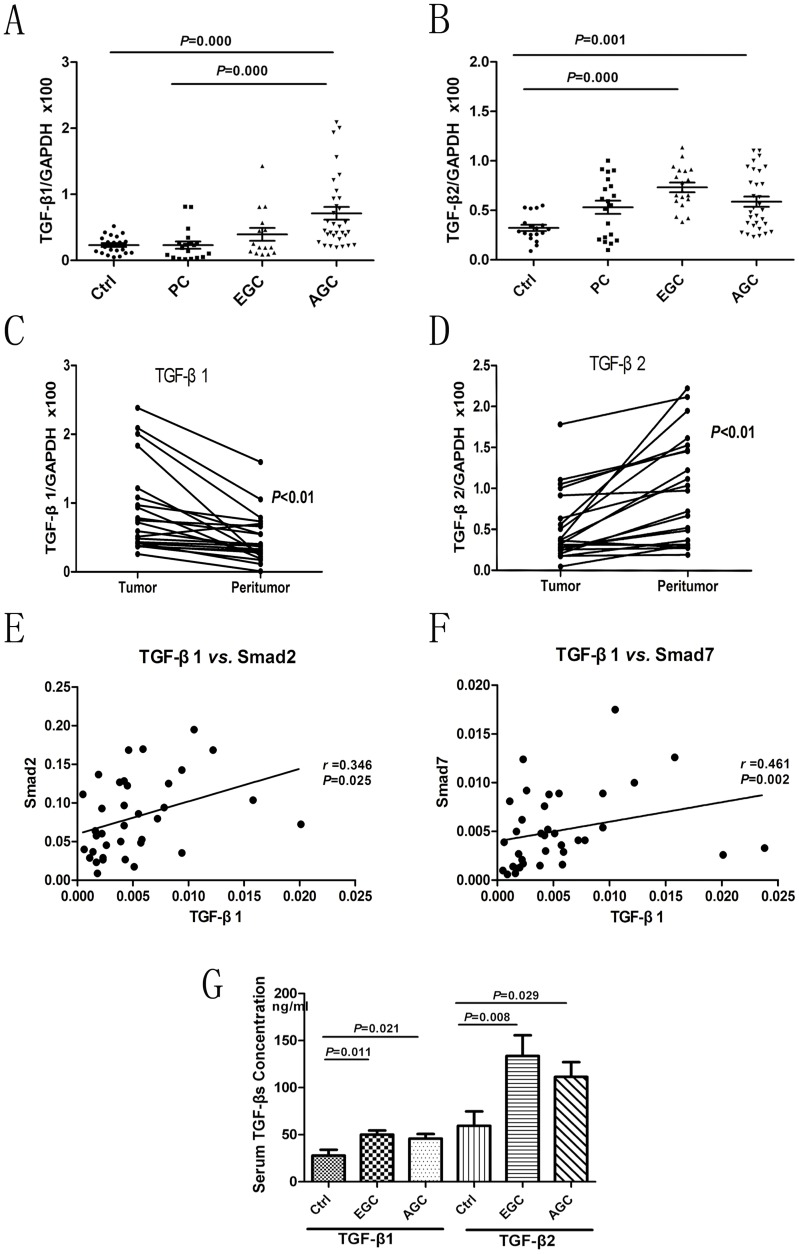
TGF-β1 and TGF-β2 mRNA profiles in gastric precancer and carcinoma. (A) TGF-β1 mRNA levels in the sequence from controls (*n* = 20), precancer (PC) (*n* = 21), early gastric cancer (EGC) (*n* = 22), to advanced gastric cancer (AGC) (*n* = 30). Data are given as means ± SD of transcript levels normalized to GAPDH. (B) Corresponding TGF-β2 mRNA levels in the same sequence. (C) and (D) TGF-β1 levels were upregulated and TGF-β2 levels were downregulated in tumor tissues, compared to peritumoral tissues from the same patients. Levels were normalized to GAPDH. Data from qRT-PCR in 20 paired cases are shown. (E) and (F) Significant positive correlations between TGF-β1 and Smad2/Smad7, using a bivariate correlation model. Data represent the transcript levels in 36 cases of GC after normalization to GAPDH. (G)Serum concentrations of TGF-β1 and TGF-β2 measured by ELISA were significantly higher in early and advanced GC compared to controls (*F = *4.745 and *P = *0.018; *F = *4.939 and *P = *0.015, respectively). There was no significant difference between early and advanced GC. *Ctrl*: controls volunteers; *EGC*: early gastric cancer; *AGC*: advanced gastric cancer.

TGF-β1 mRNA levels were increased in AGC, while TGF-β2 levels were enhanced in EGC. TGF-β1 mRNA levels increased significantly from the control, PC, EGC, and AGC stages (Figure 2A; *P*<0.05). Sub-analysis demonstrated that TGF-β1 mRNA levels were significantly higher in AGC compared to the PC and control groups (*P*<0.05), while TGF-β2 levels were increased in EGC and AGC, compared to the control group (*P*<0.01) (Figure 2B). Furthermore, TGF-β1 mRNA levels were higher in tumor than in peritumor (*P*<0.001) (Figure 2C); however, TGF-β2 levels demonstrated the opposite tendency (*P*<0.05) (Figure 2D). In addition, correlation analysis identified positive correlations between the mRNA levels of TGF-β1 and Smad2 (*r = *0.346, *P = *0.025) and Smad7 (*r = *0.461, *P = *0.002) (Figure 2E and 2F). TGF-β2, however, showed no association with Smad2 or Smad7, and neither TGF-β1 nor TGF-β2 was correlated with Smad3 or Smad4. Taken together, these results suggest that TGF-β1 and TGF-β2 might play different roles in tumor progression.

### TGF-β Serum Levels

To further explore the occurrence of TGF-β in the general environment, we compared serum concentrations of TGF-β in patients with EGC or AGC to those in controls. Serum concentrations of TGF-β1 in controls and in patients with EGC and AGC were 27.78±6.11, 50.08±4.38, and 45.76±5.00 ng/mL, respectively, while the corresponding values for TGF-β2 were 59.41±15.42, 133.61±21.90, and 111.34±15.76 ng/mL, respectively. Levels of both TGF-β1 and TGF-β2 were significantly higher in patients with EGC or AGC compared to those in controls (*F = *4.745 and *P = *0.018; *F = *4.939 and *P = *0.015). However, there were no significant differences between patients with early and late stage of GC (Figure 2G). These results suggest that the abnormal status of TGF-β in gastric carcinogenesis may be a systemic response involving not only the tumor microenvironment, but also the general circulatory system.

### Coculture In Vitro

**Figure 3:**
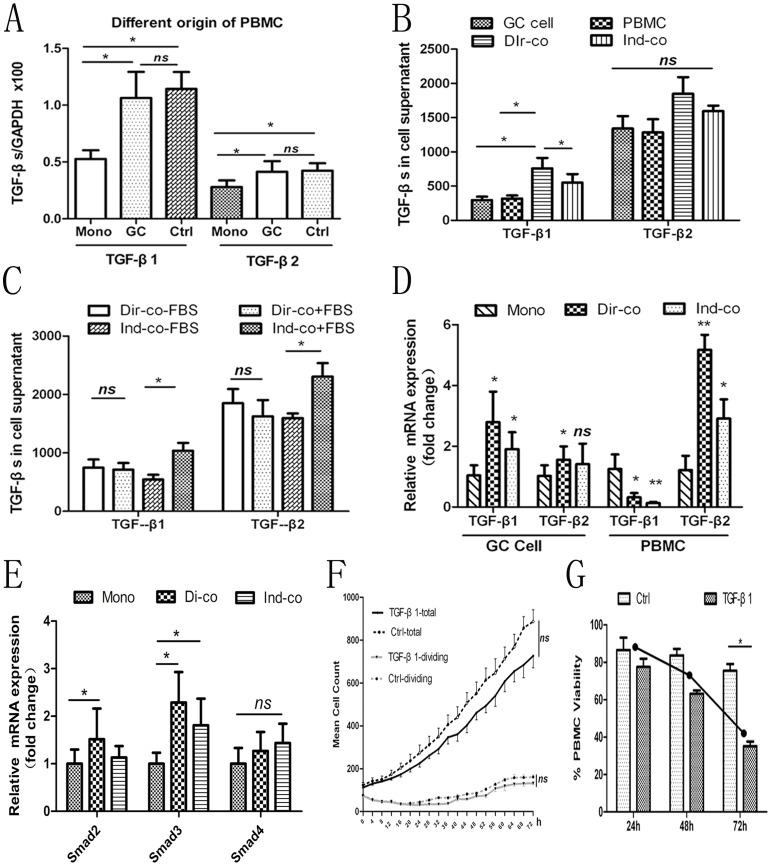
Changes in TGF-β1 and TGF-β2 expression in a coculture model. (A) TGF-β1 and TGF-β2 mRNA levels in GC cells after direct cocultures were increased compared to monoculture, but there were no significant differences in TGF-β1 and TGF-β2 mRNA levels in GC cells, irrespective of the origin of the PBMCs (GC patients or controls). (B) TGF-β1concentrations in the cell supernatant of cocultures were significantly increased compared to those in PBMCs or GCs cultured alone in a FBS-free environment (*P*<0.05). Its levels in the direct coculture group were significantly higher than those in the indirect group (*P = *0.029). TGF-β2 levels were also increased in direct cocultures, but the differences after cocultures were not significant. (C) Cytokine production levels were significantly increased in indirect coculture groups after the addition of FBS (*P*<0.05), but no obvious change was detected in direct coculture ones. The experiment was conducted twice. All data are shown as means ± SD of triplicates. (D) Origin of cytokines. In GC cells, TGF-β1 mRNA levels were increased approximately 3-fold in the direct coculture and increased 2-fold in the indirect one compared to monocultures; TGF-β2 mRNA levels were significantly increased after direct coculture but not statistically changed after indirect one. In PBMCs, TGF-β1 mRNA levels were significantly decreased and TGF-β2 levels were remarkably increased after cocultures. Levels were normalized to GAPDH, and levels in the monoculture group were defined as 1.0. All data are shown as means ± SD. (E) The mRNA levels of Smad2 and Smad3 in GC cells were significantly increased after cocultures (*P*<0.05), which were higher in the direct coculture than those in the indirect one, but there was no statistic difference in the levels of Smad4. (F) Cell-IQ showed that the addition of exogenous TGF-β1 (25 ng/mL) to GC cells suppressed the growth and division of tumor cells, but with no significant difference. Eight images from different visual field were analyzed for each group. (G) Cell Counting Kit-8 (CCK-8) assay showed that TGF-β1 (25 ng/mL) inhibited the viability of PBMCs significantly at 72 h. The line shows the inhibition ratio of TGF-β1 stimulated cells compared to untreated controls. *GC:* gastric cancer; *PMBC:* peripheral blood mononuclear cell; *Dir-co:* direct coculture; *Ind-co:* indirect coculture; *Mono:* monoculture; *FBS:* foetal bovine serum. *ns,* not significant; *, *P*<0.05; **, *P*<0.05.

A coculture model was established to determine if direct cell-to-cell contact or indirect cytokine-dependent contact is the main mechanism in a mimicking tumor microenvironment. Firstly, there were no significant difference in the results of TGF-β1 and TGF-β2 mRNA levels in GC cells in direct coculture model using PBMCs isolated from GC patients or controls (Figure 3A), and these data were therefore pooled for analysis. Furthermore, concentrations of TGF-β1 in the cell supernatant of cocultures were significantly increased compared to those in PBMCs or GCs cultured alone in a FBS-free environment (*P*<0.05) and its levels in the direct coculture group were significantly higher than those in the indirect group (*P = *0.029); however, although TGF-β2 levels were also increased in direct cocultures, the differences after cocultures were not significant (Figure 3B).

We subsequently investigated the effect of serum on the interaction between tumor cells and PBMCs. Surprisingly, TGF-β1 and TGF-β2 concentrations in the indirect group, compared to that in the FBS-free condition, were inversely higher than those in the direct group after the addition of FBS. Moreover, the concentrations of TGF-β1 and TGF-β2 in the cell supernatant were significantly increased in indirect groups (*P*<0.05), but they were only slightly increased in direct groups (*P>*0.05), by the addition of FBS (Figure 3C). This suggests that an enriched environment may facilitate cytokine production in indirect not in direct communication.

Further, to determine the origins of the cytokines, TGF-β1 and TGF-β2 mRNA levels were measured in GC cells and PBMCs respectively. Compared to monoculture, TGF-β1 mRNA levels were increased approximately 3-fold in the direct group and 2-fold in the indirect group in GC cells after coculture with PBMCs; TGF-β2 mRNA levels were significantly increased in GC cells after direct coculture but not statistically changed after indirect coculture. Meanwhile, TGF-β1 mRNA levels were decreased significantly and TGF-β2 mRNA levels were increased more than 5-fold in PBMCs after cocultures (*P*<0.05) (Figure 3D). These results indicate that the elevated TGF-β1 levels in the cell supernatant might originate from GC cells, while TGF-β2 might originate from PBMCs. In addition, we found that the mRNA levels of Smad2 and Smad3 in GC cells were also increased significantly after cocultures, which were higher in the direct coculture than those in the indirect one, but there was no statistic difference in the levels of Smad4 (Figure 3E). Overall, these results suggest that cytokines production principally depends on the direct interaction between cancer cells and PBMCs, and TGF-β/Smad2/3 signaling might be promoted during this process.

Finally, to further explore TGF-β1 roles in GC cells and PBMCs, the cell growth ability of the two types of cells were detected by adding exogenous TGF-β1 to monocultures of PBMCs or GC cells. Cell-IQ showed that the mean counts of both total and dividing cells were decreased; reconstituted TGF-β1 inhibited the growth of GC cells at 72 h, but the difference was not significant (Figure 3F), while exogenous TGF-β1 significantly affected the viability of PBMCs (Figure 3G). This finding indicates that elevated TGF-β1 mainly inhibited the function of mononuclear cells, but not of tumor cells.

Taken together, these findings in the section demonstrated that the interaction between tumor cells and PBMCs occurs principally via direct cell-to-cell contact, but with some contribution from cytokine-dependent contact. Furthermore, enriched conditions promote the production of cytokines such as TGF-β1 and TGF-β2. TGF-β1 acted mainly by inhibiting the function of PBMCs but not of GC cells.

## Discussion

Abnormalities in growth factor and cytokine secretion, especially TGF-β, play key roles in cancer development [3], [32]. However, to the best of our knowledge, the protein and mRNA statuses of TGF-β1 and TGF-β2 in precancer have not been well studied, and the production of cytokines during the interaction between tumor cells and PBMCs remains unclear. The current study therefore determined the profiles of TGF-β1 and TGF-β2 in gastric precancer and cancer, and explored the nature of the interaction (direct or indirect) between cancer cells and PBMCs and its effect on cytokine production.

Previous studies found that increased expression levels of TGF-β1 and TGF-β2 proteins were associated with poorer prognosis [5], [11], and TGF-β1 mRNA and protein levels were increased in dysplastic and GC tissues compared to normal gastric tissues [33], [34]; in contrast, TGF-β2 mRNA levels in GC tissues were comparable to controls [33]. The results of the current study confirmed and extended the earlier observations; TGF-β1 mRNA levels were significantly higher in AGC, while TGF-β2 levels were higher in dysplasia and EGC. Furthermore, TGF-β1 mRNA levels were higher in tumor than in peritumor, whereas TGF-β2 demonstrated the opposite tendency. TGF-β1 protein levels showed by IHC were consistent with these results. These findings suggest that neoplastic transformation might be an early event involving the increase of TGF-β1, together with the loss of TGF-β2.

Although a previous study demonstrated that 80% of intestinal-type GC specimens expressed TGF-β1 compared to only 43% of diffuse-type [10], we found no difference in the expression of TGF-β1 in relation to *Hp* infection, Lauren’s classification, or lymph node involvement. Activated TGF-β1 was abundant in the mucosa of *Hp*-infected patients, however, there was no significant difference compared to *Hp*-negative patients [35]. In addition, our results also showed that some stromal cells were stained positive for TGF-β1. Similarly, mononuclear cells in lamina propria were reported to be the major source of TGF-β1 [36]. Comerci *et al* [37] revealed that TGF-β1 secreted into or produced by supporting stromal elements might indirectly promote tumor progression. Ottaviano *et al* [38] showed that the crosstalk between cancer cells and stromal elements mediated by TGF-β1 influenced cell surface- and pericellular matrix-degrading potential *in vitro*. We therefore conclude that the secretion of TGF-β by tumor cells and stromal cells might play important roles in occurring and maintaining of tumor microenvironment.

The results revealed that TGF-β was also pronounced in the peripheral system, since the serum concentrations of TGF-β1 and TGF-β2 in GC patients were higher than those in controls. However, the relationship between serum concentrations of TGF-β1 and clinicopathological characters is controversial. Previous studies found that serum concentrations of TGF-β1 in GC patients were significantly higher than those in controls, and were positively correlated to tumor mass, invasion, metastasis, and clinical stage [39], [40]. Other studies, however, have reported no differences in serum TGF-β1 levels in terms of serosal involvement, lymph node involvement, vascular invasion, distant metastasis, tumor size, or histopathological grades in gastric and colon cancer [41]. Similarly, our data demonstrated no significant differences in serum TGF-β1 and TGF-β2 levels between patients with early or advanced GC. However, the release of TGF-β1 and TGF-β2 may be an early event in tumor development, since their levels were significantly increased in patients with early cancer compared to controls. Another report demonstrated that the circulating TGF-β1 levels were increased in severe dysplasia and progressed with tumor progression, and that plasma TGF-β1 activation was associated with urokinase activity resulting in the transformation of resident fibroblasts to tumor-promoting myofibroblasts [42]. Different activators thus might be involved in different tumor microenvironments, which should be explored in future studies.

The interaction between cancer cells and PBMCs is very complicate. Nowak *et al* [22] revealed that the production of TGF-β1, IL-6 and IL-10 was enhanced as a result of the interaction between PBMCs and ovarian cells. Bessler *et al* [26] showed that the production of some anti-inflammatory cytokines, such as TNF-α, IL-1β and IFN-γ was more pronounced following incubation of PBMCs with colon cancer cells, compared to that secreted by PBMC exposed to their supernatants. However, our mimicked model is a real-time coculture system, which is more comparable than the previous ones. We found that the concentrations of TGF-β cytokines were significantly increased after coculture with PBMCs compared to those when GC cells or PBMCs cultured alone, and they were higher in the direct coculture than those in the indirect one. Moreover, TGF-β1 secretion can facilitate the occurring of regulatory T cells from naïve T cells when they were cocultured with cancer cells [23]–[25]. We therefore suggest that the interaction between GC cells and PBMCs depends mainly on direct cell-to-cell contact, involving not only cytokine production but also cell differentiation.

The current study produced two other striking results. Firstly, cytokines were mostly secreted by cancer cells, since TGF-β1 mRNA levels in GC cells were up to 3-fold higher in coculture than in monoculture, while levels in PBMCs were decreased. In addition, TGF-β1 concentrations in the direct coculture group were higher than those in the indirect one. This finding supports the hypothesis that sensitized tumor cells require a constant PBMC-derived stimulus to maintain high TGF-β1 mRNA expression, and a tumor-cell-derived stimulus trigger the promotion of TGF-β2 expression in PBMCs through a cell-to-cell contact manner. Secondly, the concentrations of TGF-β1 and TGF-β2 in the indirect coculture group increased with the addition of FBS, suggesting that tumor cells can also be sensitized by PBMCs and further trigger the overexpression of TGF-β through enhancing the nutrition supply, regardless of the existence of direct physical contact with tumor cells. However, further studies are needed to determine if TGF-β itself is the sensitizing/triggering factor, or if other, as-yet undefined factors are involved.

TGF-β1 could induce growth inhibition in epithelial cells and was known to transduce intracellular signals in a Smad-dependent or -independent manner [43]. Specific inhibition of Smad pathway can suppress cancer progression by shifting Smad-dependent signaling from oncogenesis to tumor suppression [3], [44]. The current results revealed that aberrant TGF-β1 was associated with Smad2 and Smad7 expression in tumor tissues, and that direct coculture GC cells with PBMCs could promote the expression of Smad2 and Smad3. This suggests that a Smad-dependent mechanism might be existed in gastric tumor microenvironment. Moreover, exogenous TGF-β1 could reduce the viability of PBMCs, but had little influence on the growth and death of cancer cells. It might be due that cancer cell itself can increase some molecules to antagonize TGF-β1 growth-inhibitory response. As previous study reported, malignant cells can interfere TGF-β1 growth-inhibitory function and enhance cell migration through regulation of Smad2 and Smad3 activation [45]–[47]. However, TGF-β1 may arrest the growth of PBMCs and multiply immune cells by inhibiting cytokine production [2], [48]. The current study suggests that increased TGF-β1 levels in the cell supernatant of coculture systems acted mostly through inhibiting the effect of PBMCs but not of cancer cells.

There are a few limitations in this primary study: increasing the number of samples can helpful to indentify TGF-β1 roles in clinical assessment; further to investigate TGF-β1 gene’s function by interfering TGF-β1 expression in GC cells as well as *in vivo* assay will help to better explain its precise mechanism in tumor carcinogenesis. However, it could be considered in the current study that lymphocytes initially aggregate in the local microenvironment and subsequently interact directly with tumor cells, triggering GC cells to secrete more TGF-β1, which in turn inhibits the function of PBMCs and promotes tumor development.

## Supporting Information

Table S1 **Primers used for real-time PCR.**
(DOCX)
