# Supplementary material for: Transforming Growth Factor-β1 and -β2 in Gastric Precancer and Cancer and Roles in Tumor-Cell Interactions with Peripheral Blood Mononuclear Cells In Vitro
Source: PLoS One. 2013 Jan 14;8(1):e54249. doi: 10.1371/journal.pone.0054249 (PMC3544811; doi:10.1371/journal.pone.0054249)
Supplement: Table S1 — Primers used for real-time PCR. (DOCX) [file pone.0054249.s001.docx]

Table S1 Primers used for real-time PCR

| Gene and primer | | Sequences(5'to 3') | Tm-value (°C) | Production (bp) |
| --- | --- | --- | --- | --- |
| [NM_002046.3](http://www.ncbi.nlm.nih.gov/entrez/viewer.cgi?val=NM_002046.3&db=Nucleotide&dopt=GenBank) | GAPDH_F | GCACCGTCAAGGCTGAGAAC | 63.3 | 138 |
|  | GAPDH_R | TGGTGAAGACGCCAGTGGA | 64.0 |  |
| NM_000660.4 | TGF-β1_F | CCCACAACGAAATCTATGACAA | 59.9 | 246 |
|  | TGF-β1_R | AAGATAACCACTCTGGCGAGTC | 59.8 |  |
| NM_001135599.2 | TGF-β2_F | ATTGCCCTCCTACAGACTTGAG | 59.8 | 188 |
|  | TGF-β2_R | CAGCACAGAAGTTGGCATTGTA | 61.2 |  |
| NM_001003652.2 | Smad2_F | AGGTATCCCATCGAAAAGGATT | 60.0 | 189 |
|  | Smad2_R | ATACTGGAGGCAAAACTGGTGT | 59.9 |  |
| [NM_005902.3](http://www.ncbi.nlm.nih.gov/entrez/viewer.fcgi?db=nucleotide&id=52352808) | Smad3_F | CCTGGAGTAAGACACGACTTCC | 59.2 | 134 |
|  | Smad3_R | TGCATGATCCACCTGCTG | 59.6 |  |
| NM_005359.5 | Smad4_F | AGGACAGAAGCCATTGAGAGAG | 60.0 | 217 |
|  | Smad4_R | GACACTGACGCAAATCAAAGAC | 59.8 |  |
| NM_005904.3 | Smad7-F | CGATGGATTTTCTCAAACCAA | 60.3 | 135 |
|  | Smad7-R | ATTCGTTCCCCCTGTTTCA | 60.2 |  |
